# Optimization of Forming Parameters in Incremental Sheet Forming of AA3003-H18 Sheets Using Taguchi Method

**DOI:** 10.3390/ma15041458

**Published:** 2022-02-16

**Authors:** Mohanraj Murugesan, Jae-Hyeong Yu, Kyu-Seok Jung, Sung-Min Cho, Krishna Singh Bhandari, Chang-Whan Lee

**Affiliations:** 1 Department of Mechanical System Design Engineering, Seoul National University of Science and Technology, Seoul 01811, Korea; mohanaero45@seoultech.ac.kr; 2 Department of Mechanical Information Engineering, Seoul National University of Science and Technology, Seoul 01811, Korea; jhyu9109@seoultech.ac.kr (J.-H.Y.); ksjung@seoultech.ac.kr (K.-S.J.); smcho@seoultech.ac.kr (S.-M.C.); 3 Department of Mechanical Engineering, Jeju National University, Jeju-si 63243, Korea; krishna.bhandari51@gmail.com

**Keywords:** incremental sheet forming, surface finish, Taguchi L16 orthogonal array, surface roughness, ANOVA, response surface methodology

## Abstract

The surface finish is an important characteristic in the incremental sheet forming (ISF) process and is often influenced by numerous factors within the forming process. Therefore, this research was aimed at identifying the optimal forming parameters through the Taguchi method to produce high-quality formed products. The forming tool radius, spindle speed, vertical step increment, and feed rate were chosen as forming parameters in the experimental design, with surface roughness as the response variable. Taguchi L16 orthogonal array design and analysis of variance (ANOVA) test were used to identify the parameter’s optimal settings and examine the statistically significant parameters on the response, respectively. Results confirmed that a significant reduction in surface roughness occurred with a drop in vertical step size and an increase in feed rate. In detail, the vertical step size has the most significant influence on the surface roughness, followed by the feed rate and the forming tool radius. In conclusion, the optimum level settings were obtained: forming tool radius at level 3, spindle speed at level 1, vertical step size at level 1, and feed rate at level 4. Additionally, confirmation experiment results based on the optimal settings indicated a good agreement against the experimental observation. Further, the response surface methodology (RSM) was also exploited to devise a mathematical model for predicting the surface roughness. The results comparison confirmed that both techniques could effectively improvise the surface finish.

## 1. Introduction

The incremental sheet forming process (ISF) technique, also known as dieless forming, is regarded as one of the flexible manufacturing technologies due to its ability to produce complicated components without using additional dies [1,2,3,4,5]. In detail, the forming process can be performed with the help of a predesigned hemispherical-end forming tool by designing the toolpath, which defines the tool motion based on the desired profiles that need to be manufactured. Due to aforementioned advantages, this process is widely adopted in industrial applications such as medical, automotive, aerospace, and civil sectors [1,2,3,4,5]. However, although this process has noticeable advantages, it also tends to have drawbacks [6,7,8,9,10], including the material formability [11] issues such as achieving proper height and wall angle, controlling thinning behavior, reducing forming force [12,13,14], and the pillow effect. Nevertheless, the significant demand still rises in this process to obtain an excellent surface finish on the incrementally formed parts. Therefore, many researchers have carried out considerable research to identify the working parameters that control achieving better surface quality [15,16,17,18,19,20]. Correspondingly, researchers [21,22,23,24,25] have also successfully exploited the Taguchi method for many engineering applications such as Fenton process optimization [21], injection molding process [22], turning process [23], laser machining process [24], electrical discharge machining process [25], and ISF process [26,27,28,29,30,31,32,33].

The main process parameters that impact forming are tool radius, sheet thickness, step size, spindle speed, feed rate, lubrication, and toolpath selection [26,27,28,29,30,31,32,33]. For example, Kumar and Gulati [26] investigated the process for producing better surface quality using the Taguchi method by optimizing the input factors on the roughness. They concluded that the punch tool diameter was the most influencing factor for the conical frustums, followed by tool shape and viscosity level. Gulati et al. [27] revealed that a proper selection of lubrication for a tool–sheet interaction helps to enhance the surface quality. They also discussed that tool rotation and feed rate influenced the surface quality; however, sheet thickness, step size, and tool radius moderately impacted the response. Kim and Park [28] and Strano et al. [29] discussed that the material formability had some positive and negative impacts when the friction quantity was adjusted from lower to higher at certain margins. Likewise, Zhang et al. [30] also detailed the importance of lubrication selection during warm forming conditions and suggested that solid lubrication performs well to achieve good surface quality.

Similarly, the forming process was examined considering the control factors, tool radius, step size, spindle speed, and feed rate for improvising the parts surface finish by Echrif and Hrairi [31]. Echrif and Hrairi [31] have established that the forming tool radius helps to improve the surface quality more than that of other considered parameters. In addition, the feed rate has contributed less impact compared to step size and spindle speed factors. Moreover, Durante et al. [32] have indicated no considerable variation on the roughness concerning tool motion, whereas there was also no influence due to tool speed and their direction of motion. However, Radu et al. [33] demonstrated that the process parameter, spindle speed, significantly impacts the surface quality. Regardless, the studies confirm that the forming parameters contribute to the surface roughness based on individual problems assuming parts profile, machine capability, and parameter’s working range. In addition, most studies have researched the surface finish considering only simple shapes, which have linear forming regions, like a cone, or pyramid geometries. Thus, in this research, the variable hyperbolic cone profile was considered by limiting the working range of forming parameters, such as tool radius and step size, to evaluate their influence on the material formability and the surface quality.

In the present research work, the influence of four forming parameters, such as forming tool radius with two different shapes, spindle speed, vertical step-down size, and feed rate, has been examined to achieve better surface quality by minimizing surface roughness on the formed parts of AA3003-H18 Al alloy sheets. First, the experimental design was modeled using the Taguchi orthogonal L16 design based on input factors and their level settings. Then, the surface roughness was measured on the formed parts using the surface roughness tester. Further, the statistical analysis of variance (ANOVA) was conducted to recognize the forming parameters, which dominantly impact the response and their levels for achieving the minimum surface roughness. In addition, the surface roughness prediction model was proposed using the response surface methodology (RSM) according to the design table obtained from the Taguchi design and discussed using numerical and graphical verification.

## 2. Experimental Procedures

**Figure 1:**
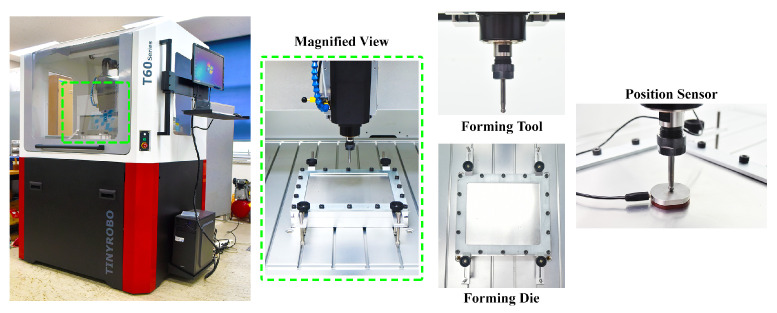
Experimental setup of single-point incremental forming process.

The material sheet used in this research is the AA3003-H18 aluminum alloy. The sheet material chemical compositions in wt % are as follows: 96.8–99.0% Al, 0.6% Si, 0.7% Fe, 0.05–0.20% Cu, 1.0–1.5% Mn, and 0.10% Zn, and the energy-dispersive X-ray spectroscopy (EDS) analysis conducted employing the field emission scanning electron microscopy method (FESEM-EDS) provides information about the presence of chemical elements present in the material [1]. Further, the test samples were prepared at 0${}^{\circ }$, 45${}^{\circ }$, and 90${}^{\circ }$ to the rolling directions to assess the AA3003-H18 Al alloy material properties such as Young’s modulus, tensile strength, yield strength, and percentage of total elongation. According to the ASTM-E8 standard, the tensile test coupled with the digital image correlation (DIC) technique was carried out using the samples prepared with 50 mm gauge length, 1.0 mm thickness, and 50×12.5 mm${}^{2}$ sample gauge area. The yield strength at 0${}^{\circ }$, 45${}^{\circ }$, and 90${}^{\circ }$ is measured as 167.47 MPa, 164.09 MPa, and 190.52 MPa, respectively, whereas the tensile strength at 0${}^{\circ }$, 45${}^{\circ }$, and 90${}^{\circ }$ is determined as 205.85 MPa, 192.88 MPa, and 214.56 MPa, respectively. Similarly, the average Young’s modulus of AA3003-H18 Al alloy material is estimated to be 69.58 MPa with the total average elongation of 5.46%. In this research work, the variable hyperbolic cone geometry is aimed to be incrementally formed with a curvature radius of 56 mm and a forming depth of 37 mm. Additionally, limiting the forming depth to 37 mm is because of the sheet thickness and fracture experienced on the tested samples during the trial runs. The sheet blank dimension used in the SPIF experiment is 280×320 mm${}^{2}$ with a thickness of 0.50 mm. The forming punch tool was fabricated from the high-speed steel (HSS) material, mostly used in cutting tools and metal forming applications, because of its material properties, such as maintaining high hardness at elevated temperatures, wear resistance, and heat resistance [1]. A schematic representation of the SPIF process experimental setup is illustrated in Figure 1; as represented in Figure 1, it has the necessary equipment such as a position sensor, a hemispherical end forming tool, a blank holder, and an external die.

## 3. Experimental Design Using Taguchi Method

**Figure 2:**
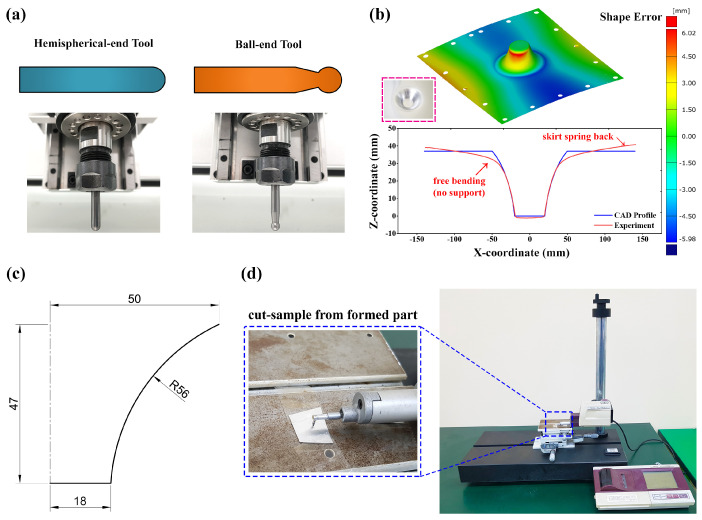
(**a**) Two kinds of forming tools used in the SPIF experiment; (**b**) scanned profile and CAD profile comparison against experiment; (**c**) 2D desired profile; (**d**) surface roughness measurement.

The forming parameters which impact the incrementally formed part surface quality, such as punch tool radius with two different shapes, spindle speed, vertical step-down size, and feed rate on the surface roughness, is considered in this research work. Table 1 includes details about the selected forming parameters and their level settings. In detail, the four-level settings forming parameters, such as step size (0.10 mm–0.25 mm), spindle speed (3000 rpm–6000 rpm), forming tool radius (2.5 mm–3.0 mm), and feed rate (500 mm/min–2000 mm/min), were used. Using this information, from available Taguchi designs, the L16 orthogonal array was selected, and the independent variables were assigned to corresponding columns as tabulated in Table 2. The vertical milling machine (Tiny CNC-6060C), which has the maximum capability of 24,000 rpm spindle speed, 12 m/min feed rate, and 600×600×200 $m^{3}$m working space, has been utilized for performing the SPIF experiments. The toolpath design for the variable hyperbolic cone profile was carried out using Fusion 360 software and directly used as NC code for forming the desired shape, Figure 2c, in the milling machine. Out of the available toolpath types, the contour toolpath was identified to offer a better surface finish and material formability than that of other toolpath designs. Therefore, the entire test cases were carried out using the contour toolpath designs. For the lubrication selection, the test samples were examined during the initial stage using three lubricants: engine oil, grease, and oil–grease combination to quantitatively assess the influence of lubricant performance on the surface finish. The manufactured part’s surface roughness was estimated as 0.80 μm, 0.66 μm, and 0.64 μm for the lubricants such as engine oil, grease, and oil–grease, respectively. Accordingly, the lubricant mixing oil and grease was identified as the most suitable option from the comparison of surface roughness measurements [1]. Table 2 data were used to perform the real-time SPIF experiments for estimating the surface roughness, as shown in Figure 2a. Additionally, the incrementally formed parts were scanned using a 3D scanner (ATOS Core camera), and generated high-quality three-dimensional measurements were employed to make a systematic comparison against the expected CAD profile, as illustrated in Figure 2b. From Figure 2b, it can be observed that the scanned experimental profile is well in agreement with the desired shape. In addition, the formed part is noticed to have extra bending due to no support in that location, and there is skirt spring-back at the edges due to the strong bolted mount, as highlighted in Figure 2b.

**Figure 3:**
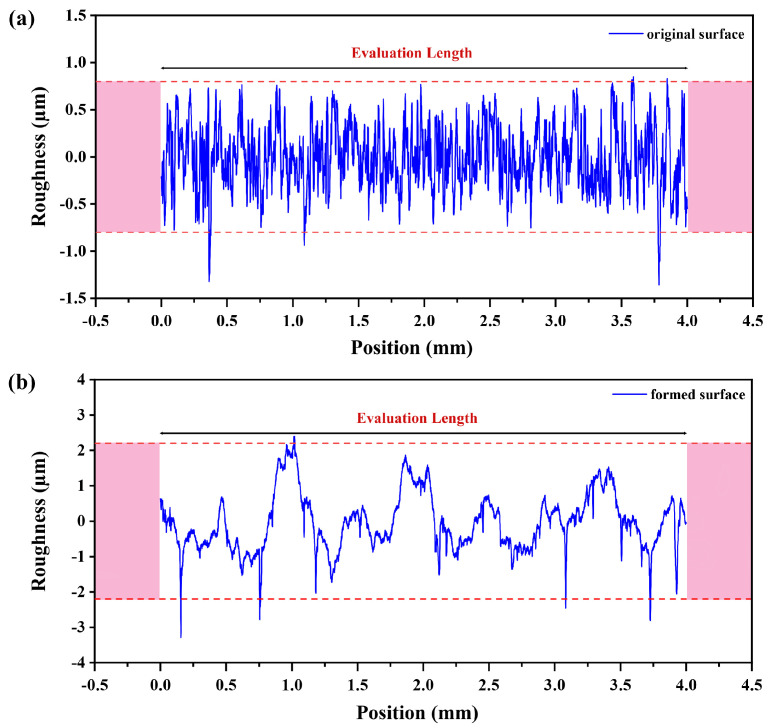
Measured surface profiles using Mitutoyo Surftest SJ-400 (**a**) original surface; (**b**) formed surface.

The surface roughness was measured in three different locations inside the formed part’s surface using the roughness tester (Mitutoyo SJ-400), as explained in Figure 2d. The measured surface roughness profiles are demonstrated in Figure 3. In detail, Figure 3a is from the original surface, where no disruption occurred, which can be observed from the uniform waviness throughout the evaluation length. In contrast, in Figure 3b, the waviness is noticed to be up and down along the evaluation length due to the punch tool motion, which causes the material deformation. The same procedures were repeated for the tested cases and computed average surface roughness values are summarized in Table 2.

**Figure 4:**
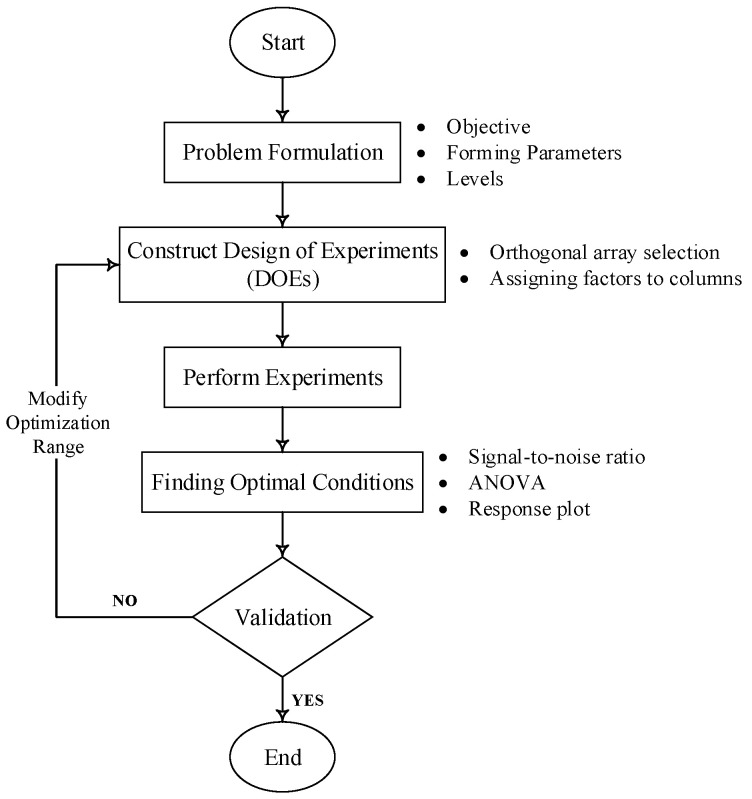
Flow chart illustrating Taguchi method procedures.

For optimizing the process parameters, Figure 4 and Table 2 details were considered to construct a roughness prediction model and identify the process parameters that affect the response, surface roughness. As this work aims to achieve better surface quality, the surface roughness has to be minimized. Thus, the S/N ratio was decided according to the smaller-the-better criterion to minimize the response. The S/N ratio is calculated using the smaller-the-better criterion, as expressed below:

(1) $$S/N\;\mathrm{ratio}=-10\times \log\frac{1}{n}\sum \left(Y^{2}\right)$$

where *Y* and *n* are responses for the given factor level combination and number of responses in the factor level combination, respectively. For assessing the influence of forming parameters on the response, the means and signal to noise (S/N) ratios for each control factor can be measured.

### 3.1. Optimization of Forming Parameters

Using designed problem descriptions and Table 2 information, the statistical toolbox, Minitab-19 licensed software, was used to calculate the signal-to-noise ratio and the means considering the smaller-the-better (Equation (1)) response. It is necessary to mention that the design problem with three process parameters was considered to have only main effects without including their interaction effects. The accomplished average surface roughness values were converted into mean and S/N ratios. The computed mean response and S/N ratio of surface roughness for each forming parameter for all levels are tabulated in Table 3. The response table of means and signal to noise ratios support recognizing the best combination of input factor-level settings that collectively optimize a selected response.

According to the highest values of the S/N ratio (Table 3), it can be concluded that minimum surface roughness could be accomplished when the vertical step-down size is smaller, the feed rate is high, and the punch tool radius is high. Based on the delta values, the process parameters rank in terms of influencing the response, surface roughness, is identified. The outcomes explain that the step-down size has a higher impact on the surface roughness, followed by feed rate and tool radius, whereas the spindle speed is noticed to affect the response moderately. Eventually, the optimum level setting was received at level 3 for tool radius, level 1 for spindle speed and step-down size, and level 4 for feed rate.

**Figure 5:**
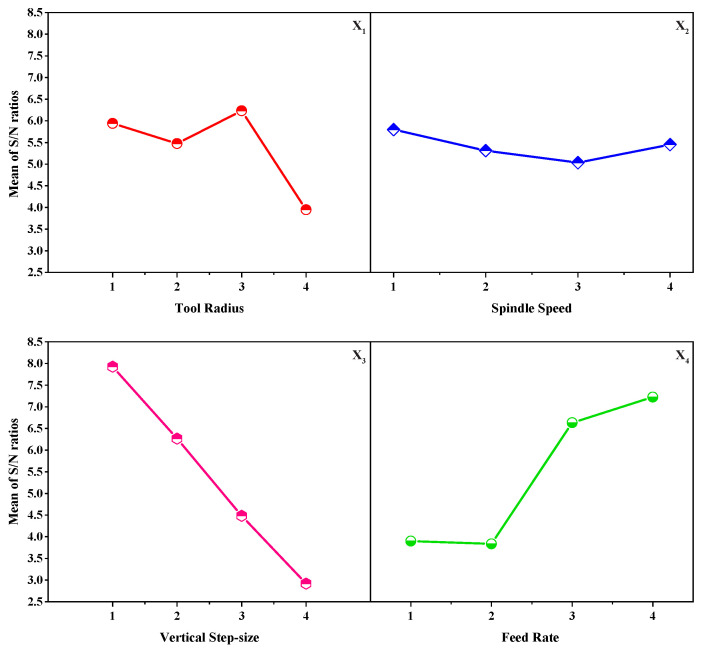
Main effects plot of S/N ratios.

Similarly, the mean values of each level control factor are used to make the main effects plot to explain the influence of forming parameters on the response. For example, the zero slope line in the main effect plot represents no considerable effect on the response, whereas the slight deflection or stepper slope defines a significant impact on the response. The estimated main effects are tabulated in Table 4, and the graphical representation of the main effects is depicted in Figure 5. Figure 5 illustrates the effect of forming parameters on the surface roughness. From Figure 5, it is observed that surface roughness decreases with a reduction in the vertical step size. The roughness reduction is because of the smooth close movement between the forming tool and the sheet blank during the forming process. Likewise, the lower surface roughness is identified at a higher feed rate; as the feed rate is higher, the forming tool motion is quick with respect to the sheet blank, and it does not stay too long at one place. In addition, the minimum surface roughness occurs when the forming radius is higher; as the forming tool radius is higher, the contact area between the tool and the blank is more eminent and causes smooth material deformation. However, the spindle speed is noticed to have a moderate effect on the response. Moreover, Figure 5 clearly explains the optimal levels of process parameters on the surface roughness.

### 3.2. Analysis of Variance (ANOVA)

The analysis of variance (ANOVA) is employed to recognize the impact of specific control input factors on the response. Furthermore, the significance level of the *p*-value was assumed to be 0.05 for determining whether the process parameters significantly affect the response. In detail, when the *p*-value is less than 0.05, the control factor substantially impacts the response and vice versa. The ANOVA statistical results for both mean and S/N ratio is computed and outlined in Table 5 and Table 6. The P-test and F-test values are estimated to be 33.42 and 0.008 for tool radius, 1.13 and 0.463 for spindle speed, 100.63 and 0.002 for step-down size, and 69.83 and 0.003 for feed rate, respectively. The outcomes of the P- and F-tests indicate that the vertical step size has the most significant influence on surface roughness, followed by feed rate, forming tool radius. In addition, the spindle speed is identified to have no significant impact on the surface roughness.

**Figure 6:**
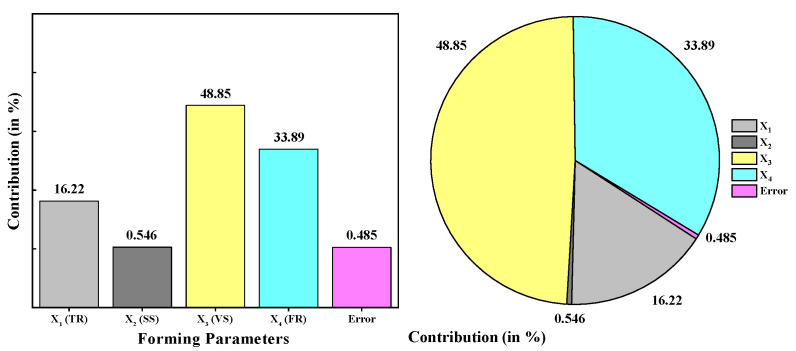
Contribution of forming parameters on measured surface roughness based on ANOVA results.

Likewise, the contribution of the forming parameters in terms of percentage is determined and detailed in Figure 6. For example, Figure 6 conveys that the vertical step-down size contributes about 49% to the response, whereas the feed rate and the tool radius contribute roughly 34% and 16%, respectively. On the other hand, the spindle speed has a contribution of approximately 0.55%, which is a nearly negligible quantity. This kind of evidence is more beneficial for selecting appropriate process parameters to improvise the response collectively.

### 3.3. Proposing Roughness Prediction Model from Optimal Settings

The optimum representation of the surface roughness is determined using the received optimal settings. The optimal level settings are identified as tool radius A3, spindle speed B1, vertical step-down size C1, and feed rate D4 with the help of the response table (Table 3) and main effect (Figure 5) and contribution (Figure 6) graphs. As a result, the optimum value of the surface roughness can be calculated as follows:

(2) $$\begin{array}{cc} \mathrm{Total}\;\mathrm{average}\;\mathrm{of}\;\mathrm{surface}\;\mathrm{roughness}\;(T) & =\sum_{i=1}^{n}\frac{y_{i}}{n}\\ \; & =0.563\;\mu m \end{array}$$

where $y_{i}$ and *n* are the surface roughness from each test condition and the total number of test cases, respectively. The optimum value of surface roughness (μ) can be computed as:

$$\begin{array}{cc} \mu & =T+\left({\bar{R}}_{\mathrm{TR}3}-T\right)+\left({\bar{R}}_{\mathrm{SS}1}-T\right)+\left({\bar{R}}_{\mathrm{VS}1}-T\right)+\left({\bar{R}}_{\mathrm{FR}4}-T\right)\\ \mu & =0.2058\;\mu m \end{array}$$

where TR3 is the average roughness at the third level of forming radius (3.0 mm), SS1 is the average roughness at the first level of spindle speed (3000 rpm), VS1 is the average roughness at the first level of vertical step size ( 0.10 mm), and FR4 is the average roughness at the fourth level of feed rate (2000 mm min^−^^1^). Eventually, the prediction based on optimal settings, signal-to-noise ratio, and the ANOVA table was utilized to model the confidence interval for the response, surface roughness. The equation used to estimate the confidence interval (CI) considering the response is represented below [15,27,34,35]:

(3) $$\mathrm{CI}=\pm \sqrt{F_{\alpha ;1;\nu_{e}}\left(\frac{1}{n_{e}}+\frac{1}{r}\right)\mathrm{MSE}}$$

In Equation (3), $\nu_{e}$ and MSE are the degrees of freedom and mean square error variance of the error, respectively. Additionally, the effective number of replications, $n_{e}={}^{N}\;{/}_{\left(\nu_{T}+1\right)}$, and *N* and $\nu_{T}$ are the total number of experiments conducted and the main factor’s degree of freedom, respectively [15,27]. The values such as $n_{e}$ and MSE from the ANOVA table (Table 5) were estimated as 1.2308 and 0.0008, respectively. Then, *F* distribution at α=0.05 was determined as 10.13 from the design table [36]. Thus, the CI was computed as below:

(4) $$\begin{array}{c} \mathrm{CI}=\pm 0.0964 \end{array}$$

Using Equation (4) information, the 95% CI for the response, surface roughness was determined as follow:

(5) 0.1094≤μ≤0.3022.

### 3.4. Confirmation Experiments

The last step in the DOE procedure is to perform confirmation tests for the optimal parameters identified. The experiments are carried out using the optimal levels, and the average surface roughness was determined to be 0.202 μm. From Table 7, it is evident that there is a good agreement between the predicted and actual data with the model error of about 1.8%. Furthermore, the confirmatory results are noticed to appear inside the CI range, which proves the model’s usefulness.

## 4. Modeling of Surface Roughness Using Response Surface Methodology

The Taguchi method contributes to reducing the number of experiments by considering the factor’s main effects and provides an optimal setting to achieve the optimum response. However, there are no modules to establish the mathematical model to explain the response against the control factors and the interaction effects among the control factors. Thus, the RSM was exploited to devise the mathematical model for the surface roughness prediction considering the process parameters such as tool radius, spindle speed, vertical step-down size, and feed rate. The RSM, often called statistical techniques for empirical modeling, is exploited to devise a mathematical model to capture the relationship between independent and response variables. It is evident that finding an appropriate function to approximate the true relationship is based on a proper experimental design. As the Taguchi method studies the control factor’s space based on the DOE fractional factorial arrays and has the advantage of handling discrete variables, the experimental design obtained from the Taguchi method can be used to develop the prediction model. A regression equation that contains more than one control factor is expressed below [1,37,38,39,40,41,42]:

(6) $$y=a_{0}+\sum_{i=1}^{k}a_{i}x_{i}+\sum_{i=1}^{k}a_{ii}x_{i}^{2}+\sum_{i=1}^{k-1}\sum_{j=1+1}^{k}a_{ij}x_{i}x_{j}+\epsilon$$

where *i* = 1, 2, …, *n*. The equation explains the presence of main and interaction effects of forming parameters. The regression model coefficients are estimated using the least squares method as follows [1,37,38,39,40,41,42]:

$$\hat{a}={\left(X^{T}X\right)}^{-1}X^{T}y.$$

Eventually, from the estimated model coefficients, $\hat{a}$, and the responses, $\hat{y}$, at unknown samples, can be calculated as [1,37,38,39,40,41,42]:

$$\hat{y}=X\hat{a}.$$

**Figure 7:**
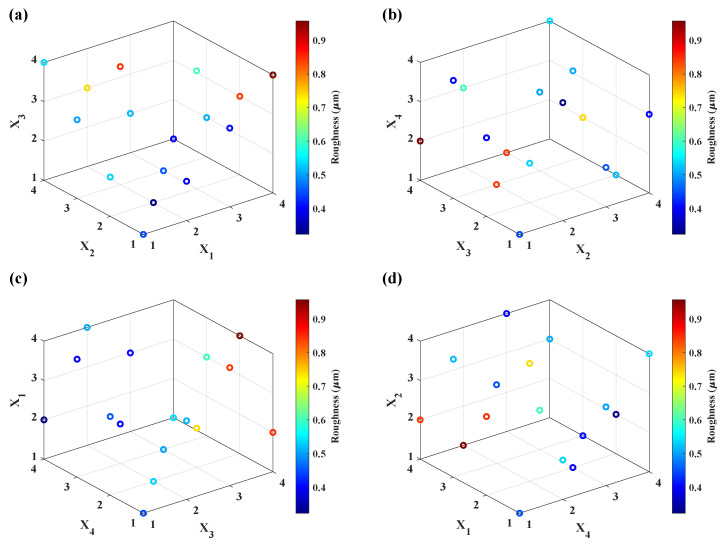
Scatter plot of forming parameters against measured surface roughness (**a**) $X_{1}X_{2}X_{3}$ vs. Roughness (μm); (**b**) $X_{2}X_{3}X_{4}$ vs. Roughness (μm); (**c**) $X_{3}X_{4}X_{1}$ vs. Roughness (μm); (**d**) $X_{4}X_{1}X_{2}$ vs. Roughness (μm).

The scatter plot reveals the presence of non-linearity in the response, surface roughness, concerning the control variables in a combination of tool radius, spindle speed, vertical step-down size, and feed rate, as represented in Figure 7. Using Figure 7a–d as a guideline, the mathematical model, Equation (10), was constructed considering the forming parameter’s main and interaction effects. The constructed surface roughness (Ra) mathematical model is expressed below:

(7) $$\begin{array}{c} {\mathrm{Ra}}_{f}=0.342-0.0045x_{1}+0.0513x_{2}+0.0028x_{3}+0.0812x_{4}\\ \;\;\;\;+0.03821x_{1}^{2}+0.00730x_{2}^{2}+0.00712x_{3}^{2}-0.0324x_{4}^{2}\\ \;\;\;\;\;\;\;\;\;\;\;\;\;\;\;-0.0798x_{1}x_{2}+0.0325x_{1}x_{4}+0.0468x_{2}x_{3}-0.04085x_{3}x_{4}. \end{array}$$

Additionally, the constructed response surface models were checked for the goodness of fit, which defines the model adequacy, using the statistical parameters such as coefficient of determination ($R^{2}$) (Equation (8)), adjusted $R^{2}$ (Equation (9)), and root mean square error (RMSE) (Equation (10)), respectively.

(8) $$R^{2}=1-\frac{\sum_{i=1}^{n}{\left(y_{e}^{i}-y_{p}^{i}\right)}^{2}}{\sum_{i=1}^{n}{\left(y_{e}^{i}-{\bar{y}}_{e}\right)}^{2}}$$

(9) $$\mathrm{Adjusted}\;R^{2}=1-\frac{{}^{\sum_{i=1}^{n}{\left(y_{e}^{i}-y_{p}^{i}\right)}^{2}}\;{/}_{\left(n-K-1\right)}}{{}^{\sum_{i=1}^{n}{\left(y_{e}^{i}-{\bar{y}}_{e}\right)}^{2}}\;{/}_{\left(n-1\right)}}$$

(10) $$\mathrm{RMSE}=\sqrt{\frac{\sum_{i=1}^{n}{\left(y_{e}^{i}-y_{p}^{i}\right)}^{2}}{n}}$$

In Equations (8)–(10), $y_{e}$, $y_{p}$, *n*, and *K* are the actual observations, the predicted observations, the total number of samples, and the number of independent variables, respectively.

**Figure 8:**
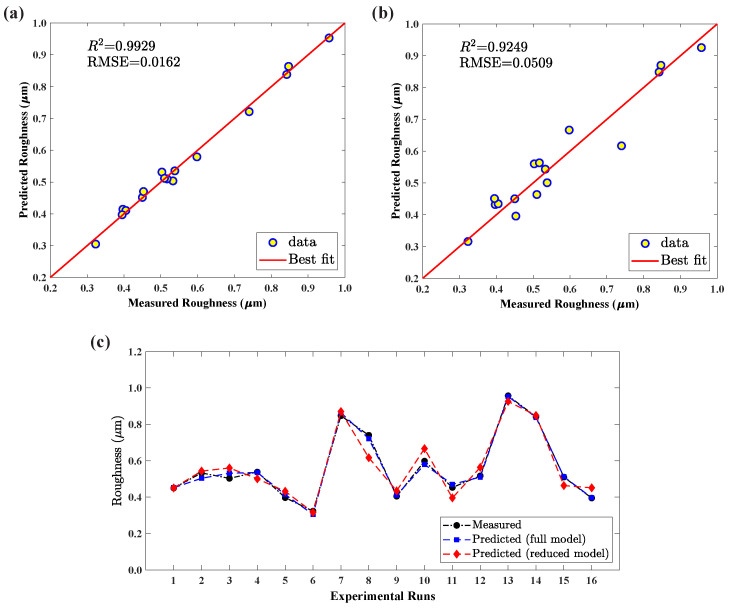
(**a**) Relationship plot of full model; (**b**) relationship plot of reduced model; (**c**) measured vs. predicted data of surface roughness.

The mathematical assessments of Equation (7) in $R^{2}$, adj.$R^{2}$, and RMSE, values were estimated to be 0.992, 0.964, and 0.0152, respectively. These numerical quantifications confirm that the second-order regression model well captured the relationship among independent and dependent variables. Figure 8a also shows that the predictions fall very close to the best fit line and prove the model’s usefulness. In addition, the ANOVA table for surface roughness was developed for establishing the significant influence of the proposed mathematical model, as outlined in Table 8. Table 8 confirms with a *p*-value less than 0.05 that the regression model has captured the response significantly considering the forming parameters. Somehow, the main factors at first-order are noticed to have no impact on the response, whereas the second-order main effects substantially affect the response. Similarly, there is a presence of interaction effects of the forming parameters on the response, as summarized in Table 8. This evidence reports that the spindle could not be skipped from selected forming parameters. However, the confirmation can be accomplished by proposing another regression model without considering the spindle speed parameter.

Therefore, using the aforementioned discussions and Figure 5 information from the Taguchi method, the constructed regression model, Equation (7), was modified by omitting the spindle speed parameter, and the reduced model is shown below:

(11) $$\begin{array}{c} {\mathrm{Ra}}_{r}=0.424-0.180x_{1}+0.136x_{3}+0.062x_{4}+0.0371x_{1}^{2}+0.0071x_{3}^{2}-0.0090x_{4}^{2}\\ +0.0122x_{1}x_{3}-0.0029x_{1}x_{4}-0.0362x_{3}x_{4} \end{array}$$

Similarly, the statistical parameters of Equation (11), $R^{2}$, adj.$R^{2}$, and RMSE, were computed as 0.925, 0.812, and 0.0494, respectively. This outcome proves that the model reduction does not improve the model performance but somehow affects its prediction capability. Apart from the numerical verification, graphical representations such as relationship and comparison plots are materialized, as shown in Figure 8. The correlation plot, Figure 8a, explains the stronger relationship between actual and predicted data acquired from the full model compared to the reduced model, as shown in Figure 8b. Additionally, Figure 8c conveys that the surface roughness predictions from the full model agree well with the experimental observations, better than that of the predictions from the reduced model. The discussions ensure that the spindle speed could not be omitted from the consideration. Correspondingly, A. Kumar et al. [26] also discussed that the improvement in the surface quality happened when the forming tool motion was changed from free-to-rotate to 1000 rpm. The improvement occurred because of the reduction in tool marking due to the contact and caused smooth movement between tool and sheet. These outcomes establish that the proposed mathematical model could be used for surface roughness optimization and predict the surface roughness at the unknown random samples in the design space.

## 5. Conclusions

The effects of forming parameters on the surface roughness of incrementally formed parts have been studied experimentally on AA3003-H18 Al alloy sheets. Then, the process parameters were examined and optimized using the Taguchi technique considering the average surface roughness on the AA3003-H18 alloy sheet. Preliminary investigation results showed that better surface quality (Ra = 0.64 μm) was obtained using the combination of engine oil and grease lubricant, whereas low surface quality (Ra = 0.80 μm) was received from the engine oil lubricant. The lubricant selection has been confirmed to be a significant factor for increasing the surface finish of formed parts. The following findings were made based on the Taguchi method outcome:

1. The Taguchi L16 orthogonal array design has contributed better surface finish at the sixth test run, and the surface roughness was calculated to be 0.323 μm.
2. Taguchi results revealed that the optimum level setting was received for obtaining minimum surface roughness at 3.0 mm of forming tool radius, 3000 rpm of spindle speed, 0.10 mm of vertical step size, and 2000 mm/min of feed rate.
3. Based on ANOVA results such as P-test and F-test and contribution graphs, vertical step size is the most significant factor with a contribution of 48.85%, followed by feed rate (33.89%), forming tool radius (16.22%), and spindle speed (0.546%).
4. The formed part’s surface roughness increased at high vertical step increment and low feed rate, whereas it decreased with the increase in feed rate and tool radius and the decrease in vertical step size.
5. Confirmation tests performed at optimal level settings of forming process parameters revealed that surface roughness was within the confidence interval at 95% confidence level and showed close agreement to the experimental data. Therefore, the proposed Taguchi technique efficiently predicted the formed part’s surface roughness.
6. Furthermore, the proposed mathematical model results considering the input factor’s main and interaction effects agreed well with the experimental observations.

Thus, the SPIF procedures devised in this research work can be exploited for various materials to enhance the surface quality or help implement the forming process at the industrial scale.

## Figures and Tables

**Table 1:** Input forming parameters and their level settings.

Parameters	Levels			
	A	B	C	D
Tool Radius (TR) (in mm)	R2.5	R3.0	T3.0	T2.5
Spindle Speed (SS) (in rpm)	3000	4000	5000	6000
Vertical Step Size (VS) (in mm)	0.10	0.15	0.20	0.25
Feed Rate (FR) (in mm/min)	500	1000	1500	2000

**Table 2:** Experimental design from Taguchi L16 orthogonal array.

Runs	TR (mm)	SS (rpm)	VS (mm)	FR (mm/min)	Roughness (μm)	$\mu_{N}$	S/N Ratio
1	R2.5	3000	0.10	500	0.450	0.200	6.936
2	R2.5	4000	0.15	1000	0.533	0.331	5.465
3	R2.5	5000	0.20	1500	0.503	0.284	5.969
4	R2.5	6000	0.25	2000	0.538	0.339	5.384
5	R3.0	3000	0.15	1500	0.397	0.117	8.024
6	R3.0	4000	0.10	2000	0.323	0.000	9.816
7	R3.0	5000	0.25	500	0.847	0.826	1.442
8	R3.0	6000	0.20	1000	0.740	0.658	2.615
9	T3.0	3000	0.20	2000	0.405	0.129	7.851
10	T3.0	4000	0.25	1500	0.598	0.434	4.466
11	T3.0	5000	0.10	1000	0.453	0.205	6.878
12	T3.0	6000	0.15	500	0.517	0.306	5.730
13	T2.5	3000	0.25	1000	0.957	1.000	0.382
14	T2.5	4000	0.20	500	0.842	0.819	1.494
15	T2.5	5000	0.15	2000	0.510	0.295	5.849
16	T2.5	6000	0.10	1500	0.395	0.114	8.068

**Table 3:** Response table for means and S/N ratios.

Levels	Means				S/N Ratio			
	A	B	C	D	A	B	C	D
1	0.5060	**0.5523**	**0.4053**	0.6640	5.939	**5.798**	**7.924**	3.901
2	0.5767	0.5740	0.4893	0.6708	5.474	5.310	6.267	3.835
3	**0.4933**	0.5783	0.6225	0.4733	**6.231**	5.034	4.482	6.632
4	0.6760	0.5475	0.7350	**0.4440**	3.948	5.449	2.919	**7.225**
**Delta**	0.1827	0.0308	0.3297	0.2268	2.283	0.764	5.006	3.390
**Rank**	3	4	1	2	3	4	1	2

**Table 4:** Main effects of forming parameters on mean S/N ratios.

$X_{1}$	S/N Ratio	$X_{2}$	S/N Ratio	$X_{3}$	S/N Ratio	$X_{4}$	S/N Ratio
1	5.939	3000	5.798	0.10	7.924	500	3.900
2	5.475	4000	5.310	0.15	6.267	1000	3.835
3	6.231	5000	5.034	0.20	4.482	1500	6.632
4	3.948	6000	5.449	0.25	2.919	2000	7.225

**Table 5:** Analysis of variance (ANOVA) table for means.

Source	DF	Adj SS	Adj MS	F-Value	*p*-Value
**A**	3	0.0843	0.0281	33.42	0.008 ${}^{*}$
**B**	3	0.0028	0.0009	1.13	0.463 ${}^{\#}$
**C**	3	0.2538	0.0846	100.63	0.002 ${}^{*}$
**D**	3	0.1761	0.0587	69.83	0.003 ${}^{*}$
**Error**	3	0.0025	0.0008		
**Total**	15	0.5195			
*—**significant** and #—**non-significant**					

**Table 6:** Analysis of variance (ANOVA) table for S/N ratios.

Source	DF	Adj SS	Adj MS	F-Value	*p*-Value
**A**	3	12.379	4.1263	10.69	0.041 ${}^{*}$
**B**	3	1.2110	0.4036	1.05	0.486 ${}^{\#}$
**C**	3	56.498	18.8326	48.78	0.005 ${}^{*}$
**D**	3	38.179	12.7264	32.96	0.009 ${}^{*}$
**Error**	3	1.1580	0.3861		
**Total**	15	109.425			
*—**significant** and #—**non-significant **					

**Table 7:** Optimal forming settings from Taguchi method and confirmation results.

Settings				Prediction		Confirmation Experiment	
$X_{1}$	$X_{2}$	$X_{3}$	$X_{4}$	**S/N Ratio**	**Mean**	**Mean**	**Error (%)**
3	1	1	4	10.9846	0.20575	0.202	1.8
T3.0	3000 rpm	0.10 mm	2000 mm/min				

**Table 8:** Analysis of variance (ANOVA) table for surface roughness.

Source	DF	Adj SS	Adj MS	F-Value	*p*-Value
Regression	12	0.515844	0.042987	34.80	**0.007**
$X_{1}$	1	0.000007	0.000007	0.01	0.943
$X_{2}$	1	0.000530	0.000530	0.43	0.559
$X_{3}$	1	0.000002	0.000002	0.00	0.967
$X_{4}$	1	0.001616	0.001616	1.31	0.336
$X_{1}$*$X_{1}$	1	0.022041	0.022041	17.84	0.024
$X_{2}$*$X_{2}$	1	0.000688	0.000688	0.56	0.510
$X_{3}$*$X_{3}$	1	0.000812	0.000812	0.66	0.477
$X_{4}$*$X_{4}$	1	0.009142	0.009142	7.40	0.073
$X_{1}$*$X_{2}$	1	0.027447	0.027447	22.22	0.018
$X_{1}$*$X_{4}$	1	0.011976	0.011976	9.69	0.053
$X_{2}$*$X_{3}$	1	0.010452	0.010452	8.46	0.062
$X_{3}$*$X_{4}$	1	0.027812	0.027812	22.51	0.018
Error	3	0.003706	0.001235		
Total	15	0.519550			

## Data Availability

Not applicable.

## Abbreviations

The following abbreviations are used in this manuscript:

CNC	computer numerical control
ISF	incremental sheet forming process
SPIF	single-point incremental forming process
DOE	design of experiments
$\mu_{N}$	normalized surface roughness
$R^{2}$	coefficient of determination
RMSE	root mean square error
RSM	response surface methodology
ANOVA	statistical analysis of variance
CAD	computer-aided design
CI	confidence interval
MSE	mean square error
DF	degrees of freedom
Adj MS	adjusted mean squares
Adj SS	adjusted sum of squares
